# How effective is collagen resorbable membrane placement after partially impacted mandibular third molar surgery on postoperative morbidity? A prospective randomized comparative study

**DOI:** 10.1186/s12903-017-0416-z

**Published:** 2017-10-05

**Authors:** Adnan Kilinc, Mert Ataol

**Affiliations:** Department of Oral and Maxillofacial Surgery, Faculty of Dentistry, Ataturk University, 25240 Erzurum, Turkey

**Keywords:** Collagen resorbable membrane, Mandibular third molar surgery, Partially impacted tooth, Postoperative morbidity

## Abstract

**Background:**

Collagen membranes have some benefits include promoting wound healing through isolation, clot stabilization, wound stability, and hemostasis, enhancing primary wound coverage through its chemotactic ability to attract fibroblasts, and augmenting flap thickness by providing a collagenous scaffold. The purpose of this study was to compare primary and secondary healing and collagen membrane-based primary healing after surgical removal of partial impacted mandibular third molars, evaluating the incidence of postoperative complications and analyzing the swelling, mouth opening, and pain.

**Methods:**

This was a prospective, randomized controlled study. Patients were randomly assigned to three groups: the SC (Secondary closure) group, the PC (Primary closure) group, and the MBPC (membrane based primary closure) group. Data were collected on pain, mouth opening, swelling, and complications experienced by the patients.

**Results:**

There was no statistically significant difference between the groups for the pain (*p* > 0.05), relatively. The swelling recorded on postoperative days 2 and 7 was lower in the SC group than in the PC (*p* = 0.046 and 0.00) and in MBPC (*p* = 0.005 and 0.002) groups, respectively. Mouth opening showed a statistically significant difference between the three groups at day 2 (*p* = 0.00). Wound dehiscence was shown in 6 patients in the PC (20%) group and 2 patients in the MBPC (6.7%) group. Dry socket was observed 3 patients in the SC group (10%), 2 patients in the PC group (6.7%), and no dry socket in the MBPC group. No cases of infection or postoperative bleeding were encountered.

**Conclusions:**

The secondary closure provides a marked advantage over the primary closure in terms of swelling and mouth opening. However, the absence of alveolitis in the primary closure using the collagen membrane and minimal wound dehiscence, suggests that membrane use may support primary healing in terms of wound healing.

## Background

Mandibular third molar (3 M) surgery can be associated with different complications such as postoperative pain, swelling, trismus, alveolar osteitis, dehiscence, infection, and periodontal destruction in the adjacent second molars [1–3].

Many researchers have focused on factors affecting the postoperative complications associated with this surgery, investigating subjects, such as surgical techniques, drugs, and instruments, for minimizing these complications [4–6]. Wound closure is another subject associated with 3 M surgery, and two methods have been proposed: primary closure, in which the mucoperiosteal flap is complete and hermetically closed, and secondary closure, in which the mucoperiosteal flap is partially closed, leaving a gap created to allow healing of the wound area [1, 2]. With secondary healing, drainage has reportedly been better and postoperative pain and swelling reduced [1]. Primary closure has been suggested to enhance patient comfort and promote postoperative wound healing [7]. However, a recent systematic review and meta-analysis of randomized controlled trials has suggested that there may not be important differences in outcomes between the secondary and primary closure techniques in patients undergoing surgical removal of impacted mandibular 3Ms [8]. Hence, new research is needed to make a clearer distinction.

Furthermore when we consider partially erupted impacted molars, primary closure of the wound site is only possible with a sliding flap, and this would be more risky due to increased tension of the flap in the wound area as compared to total mucosa sealed teeth without tissue loss [3, 7]. In such cases, the use of materials that support primary wound healing may be considered.

Collagen membranes have been used in both the medical and dental fields for decades. The benefits of utilizing this absorbable material include promoting wound healing through isolation, clot stabilization, wound stability, and hemostasis, enhancing primary wound coverage through its chemotactic ability to attract fibroblasts, and augmenting flap thickness by providing a collagenous scaffold [9]. Studies examining the effects of the use of membranes after removal of impacted 3Ms have mainly focused on the periodontal health of the 2 M or the bone regenerative capacity, and the evaluation of postoperative morbidity and wound healing is lacking in this respect [10–12].

This study compared primary and secondary healing and collagen membrane-based primary healing after surgical removal of partial erupted impacted 3Ms, evaluating the incidence of postoperative complications and analyzing the extent of swelling, degree of limitation of mouth opening, and the severity of pain.By the way, we would be able to comment on whether resorbable collagene membrane usage is appropriate and preferable.

## Methods

This was a prospective, randomized controlled study and the study sample included patients with no history of medical illness or medication that could influence the course of postoperative wound healing or alter their wound healing after surgery and patients with vertical or mesioangular partially impacted 3 M with healthy periodontium or localized chronic periodontitis. Patients were excluded from randomization if they had a preexisting abscess or cellulitis, acute pericoronitis, any restorations or carious lesions on distal surfaces of 2 Ms or pathological findings associated with their 3Ms, those using contraceptives, and smokers.

Patients were randomly assigned to three groups: the SC (Secondary closure) group, involving partial closure of the extraction site to allow secondary healing; the PC (Primary closure) group, involving total closure of the extraction site by sliding the flap to allow primary healing; and the MBPC (membrane based primary closure) group, involving total closure of the extraction site by sliding the flap and using a collagen membrane. The examination and clinical selection of the patients was carried out by a single researcher who performed the surgical procedure. Patients were randomised by the envelope method. Postoperative measurements performed not by the surgeon but by the other third-party blinded researcher who had no information about the patient and study group of patient.

Surgical operations were carried out under local anesthesia, which was achieved with 4 mL of articaine and 1:100,000 epinephrine (Ultracaine D-S Forte, Aventis). Anesthesia was achieved by a combination of inferior alveolar, lingual, and buccal nerve blocks. Additional volume of anesthesia was used and properly documented when necessary. Access was gained through a full thickness mucoperiosteal triangular flap. Osteotomy was performed with the bur, and tooth sectioning was performed when necessary under constant irrigation with sterile isotonic saline. After extraction, curettage and elimination of the remaining dental follicle were performed. In all cases, wound closure was carried out with atraumatic silk sutures.

The wound closure was SC, PC, or MBPC, depending on which group the patient belonged to. In the PC group, the flap was rotated and sliding, then repositioned to allow for healing by primary intention, before it was sutured to seal off communication with the oral cavity. In the MBPC group, a resorbable collagen membrane (Evolution ®; Osteobiol-Tecnoss, Italy) was positioned to extend 3–4 mm beyond the margin of the bone defect, and the area was then closed in the same way as a total closure.

In the SC group, the flap was sutured, leaving a gap without shifting to the former position by placing a single suture distal to the 2 M, if necessary, by removing a wedge of mucosa.

The patients were given standard postoperative instructions. Patients were administered antibiotics (amoxicillin + clavulanic acid, 2 g per day for 5 days), nonsteroidal anti-inflammatory drug (dexketoprofen trometamol 25 mg, twice daily for 3 days) and mouthwash (with 0.12% chlorhexidine twice daily for 7 days). The sutures were removed after 7 days.

Pain was evaluated by the patients on a daily basis for 7 postoperative days using a visual analog scale (VAS) data sheet with a score of 0 to 10. The patients were advised to evaluate for pain using the VAS and to record the score on the data sheet. A score of 0 indicated no pain, while 10 indicated the worst pain. Swelling was assessed by both subjective and objective methods. In the subjective method, swelling was evaluated by the patient on a daily basis for 7 postoperative days using VAS data sheet with a score of 0 to 10. The patients were advised to evaluate for swelling using the VAS and to record the score on the data sheet. A score of 0 indicated no swelling, and 10 indicated the worst swelling. The scores were measured and calculated by the researcher. In the objective method, swelling was measured by a tape measuring method preoperatively and on postoperative days 2 and 7. Two measurements were taken between the tragus to lip junction and the distance between the external corner of the eye to the mandibular angle. This distance was measured using a flexible tape-measure graduated in millimeters, which was placed in contact with the skin. The measurements were repeated twice. The final value was calculated by taking the average of the obtained measurements. Swelling was considered as the difference between preoperative and post-operative measurements at the same points. Mouth opening was measured by taking the maximum distance between the maxillary and mandibular central incisors using a caliper before surgery and on days 2 and 7 postoperatively. All the measurements were repeated twice, and the data were recorded. Assessment of other complications was conducted on day 2, day 7, at 1 month, and on any other day required by the patient. The assessment was performed using set criteria described by Bello [2], and their presence or absence was properly documented. Postoperative bleeding was considered present if a patient complained of bleeding within 48 h after being discharged from the hospital. Dry socket was diagnosed if a patient presented to the clinic days after surgery with a painful and necrotic socket that was otherwise surrounded by healthy gingival tissue and there was no suppuration. Socket infection was diagnosed when the following criteria were met: history of pus discharge with or without pain, bleeding 1 week or more after surgery, and a suppurative socket with or without fever [2].

Wound dehiscence was noted on the 7th postoperative day. The wound was considered to be dehisced if there was gaping along the entire incision line. If dehiscence was found, it was noted whether infection or alveolitis was present [7].

Other parameters determined in this study were gender, duration of surgery, surgical difficulty, and the position of the 3Ms. Surgical difficulty was recorded immediately after the procedure and evaluated using a difficulty rating on a 4-class scale: 1, extraction requiring forceps only; 2, extraction requiring osteotomy; 3, extraction requiring osteotomy and coronal section; and 4, complex extraction (root section) [13]. The level of impaction for the 3Ms was classified according to Pell and Gregory’s classification: on the level of the occlusal plane or between the occlusal plane and the cervical line of the 2 M (below the occlusal plane). 3 M angulation was classified according to Winter’s classification on a 2-class scale: 1, mesioangular; and 2, vertical. Orthopantomography (OPG) was obtained to ensure the similarity of the type of impaction according to the angulation and relationship to the occlusal plane.

Statistical analysis was performed using the Statistical Package for Social Sciences statistical software package (SPSS for Windows, version 20.0, SPSS, Chicago, IL). The normalities of the distributions were tested using the Kolmogorov-Smirnov test. For the intergroup comparisons, the chi-squared test, independent 2 sample test, Kruskal-Wallis, and/or Mann-Whitney U tests were used. Descriptive statistics were computed. Significance level was set as *p* < 0.05.

## Results

Ninety patients were included in this study. There were 24 men (26. 7%) and 66 women (73.3%), giving a male-to-female ratio of 1:2.75. Each of three study groups included 30 patients. Table 1 compares the 3 groups based on factors that could affect the outcome variables, including age, gender, angulation, depth, surgical difficulty and operation time. These results show no statistically significant difference (*p* > 0.05) except operation time (*p* = 0.003) (Table 1). SC had a significantly shorter operation time than PC (*p* = 0.012) and MBPC (*p* = 0.001). There were no significant differences between the PC and MBPC groups (*p* > 0.05).

**Table 1:** Study Variables and Descriptive Statistics

	SC	PC	MBPC	
	Mean or n (Std or %)	Mean or n (Std or %)	Mean or n (Std or %)	(p)
Age	22.10	23.47	23.67	0.40
Gender				
Female	22 (73.3)	23 (76.7)	21 (70)	0.843
Male	8 (26.7)	7 (23.3)	9 (30)	
Angulation				
Vertical	21 (70)	17 (56.7)	23 (76.7)	0.241
Mesioangular	9 (30)	13 (43.3)	7 (23.3)	
Depth				
Occlusal plane	19 (63.3)	16 (53.3)	17 (56.7)	0.727
Below occlusal plane	11 (36.7)	14 (46.7)	13 (43.3)	
Surgical Difficulty				
1	15 (5)	14 (46.7)	15 (50)	0.642
2	6 (20)	5 (16.7)	7 (23.3)	
3	6 (20)	7 (23.3)	2 (6.7)	
4	3 (10)	4 (13.3)	6 (20)	
Operation Time	11.07	15.00	15.83	0.003^*^

*Std* Standard Deviation, ^*^: *p* < 0.05

**Fig. 1:**
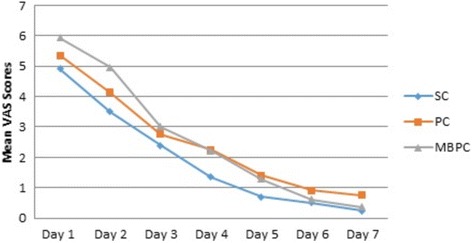
Pain VAS Scores

The pain scores were the highest on the first day in all groups, showing a gradual decrease in the following days (Fig. 1). Although the pain scores were generally slightly better in the SC group than in the PC and MBPC groups, there was no statistically significant difference between the 3 groups (*p* > 0.05) except between SC and MBPC on the second day (*p* = 0.014). Table 2 shows the mean, standard deviations, and comparison results between groups for the pain scores in all groups.

**Table 2:** Comparison of Outcome Variables of Pain, Swelling, and Mouth Opening

	SC	PC	MBPC		SC / PC	SC / MBPC	PC / MBPC
	Mean (STD)	Mean (STD)	Mean (STD)	p	p	p	p
Objective Measurements							
Mouth opening^1^ (%)							
Day 2	68.76 (1.07)	60.38 (1.88)	59.76 (3.26)	0.000^*^	0.000^*^	0.000^*^	0.029^*^
Day 7	75.82 (22.82)	68.86 (13.49)	64.49 (14.90)	0.093	0.155	0.062	0.238
Swelling^2^ (mm)							
Day 2	9.15 (4.31)	14.82 (11.05)	18.43 (16.31)	0.015^*^	0.046^*^	0.005^*^	0.403
Day 7	2.72 (1.34)	5.96 (4.66)	8.17 (10.62)	0.001^*^	0.000^*^	0.002^*^	0.656
Subjective Measurements							
Pain (VAS)							
Day 1	4.91 (3.32)	5.36 (3.01)	5.94 (2.16)	0.191	0.614	0.160	0.225
Day 2	3.51 (1.91)	4.14 (3.26)	4.97 (2.51)	0.104	0.363	0.014^*^	0.275
Day 3	2.40 (2.17)	2.76 (2.61)	3.01 (1.98)	0.294	0.455	0.131	0.363
Day 4	1.35 (1.42)	2.26 (2.87)	2.21 (2.12)	0.256	0.445	0.084	0.476
Day 5	0.70 (0.88)	1.40 (1.93)	1.27 (1.39)	0.226	0.194	0.097	0.814
Day 6	0.50 (0.75)	0.92 (1.64)	0.61 (0.86)	0.824	0.541	0.707	0.804
Day 7	0.23 (0.43)	0.75 (1.88)	0.37 (0.69)	0.892	0.734	0.627	0.969
Swelling (VAS)							
Day 1	1.67 (1.96)	3.05 (2.91)	5.80 (1.89)	0.000^*^	0.184	0.000^*^	0.000^*^
Day 2	1.91 (2.20)	4.05 (3.71)	6.82 (2.27)	0.000^*^	0.019^*^	0.000^*^	0.003^*^
Day 3	1.23 (1.54)	3.93 (3.72)	6.72 (2.22)	0.000^*^	0.004^*^	0.000^*^	0.006^*^
Day 4	0.72 (1.05)	3.10 (3.28)	5.03 (2.10)	0.000^*^	0.002^*^	0.000^*^	0.013^*^
Day 5	0.49 (0.67)	2.41 (2.55)	3.39 (1.72)	0.000^*^	0.005^*^	0.000^*^	0.037^*^
Day 6	0.16 (0.38)	1.29 (1.52)	1.99 (1.33)	0.000^*^	0.001^*^	0.000^*^	0.019^*^
Day 7	0.07 (0.18)	0.54 (0.83)	0.95 (0.95)	0.000^*^	0.015^*^	0.000^*^	0.045^*^

^*^: Significant, *p* < 0.05

^1^ Mouth opening was measured by taking the maximum distance between the maxillary and mandibular central incisors using a caliper. Mouth opening was consider as the percentage difference between the two measurements

^2^ Swelling was measured by a tape measuring method and two measurements were taken between the tragus to lip junction and the distance between the external corner of the eye to the mandibular angle. Swelling was considered as the difference between the two measurements

**Fig. 2:**
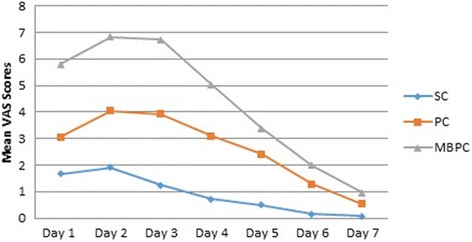
Swelling VAS Scores

In terms of swelling, there was a statistically significant difference between groups on the 2^nd^ and 7^th^ days (*p* < 0.05). The swelling recorded on postoperative days 2 and 7 was lower in the SC group than in the PC (*p* = 0.046 and 0.000) and in MBPC (*p* = 0.005 and 0.002) groups, respectively. There were no significant differences between the PC and MBPC groups (*p* > 0.05). When the swelling was assessed subjectively with VAS, the scores showed a statistically significant difference between the three groups at all times recorded (*p* < 0.05). However, there was no statistically significant difference between SC and PC on the 1^st^ day (*p* = 0.184).The peak of swelling was shown on the 2^nd^ day in all groups. Table 2 shows the mean, standard deviations, and comparison results between groups for the swelling scores and measurements in all groups (Fig. 2).

Mouth opening showed a statistically significant difference between the three groups at day 2 (*p* 0.000), although at day 7, the differences between groups were not statistically significant (*p* = 0.093). MBPC had lower statistically significant mouth opening rate on day 2 than SC (*p* = 0.029) and PC (*p* = 0.000). Additionally, PC had lower statistically significant mouth opening rate than SC (*p* = 0.000). Although the trismus scores on the 7^th^ day were generally slightly better in the SC group, the differences between the 3 groups were not statistically significant (*p* > 0.05). Table 2 shows the mean, standard deviations, and comparison results between groups for the mouth opening rate in all the groups.

The postoperative complications and comparisons between the groups are listed in Table 3. According to the results, wound dehiscence was shown in 6 patients in the PC (20%) group and 2 patients in the MBPC (6.7%) group at the check-up on postoperative day 7. There was no statistically significant difference between these groups (*p* = 0.129). Dry socket was observed in 2 dehiscence cases of the PC group. Dry socket was observed 3 patients in the SC group (10%), 2 patients in the PC group (6.7%), and no dry socket in the MBPC group, and the differences between the groups were not statistically significant (*p* > 0.05). All the cases could be treated by local measures. No cases of infection or postoperative bleeding were encountered.

**Table 3:** Postoperative complications

	SC	PC	MBPC	
	n (%)	n (%)	n (%)	P
Dehiscence	–	6 (20)	2 (6.7)	0.129
Dry Socket	3 (10)	2 (6.7)	0	0.227
Infection	0	0	0	–
Bleeding	0	0	0	–

## Discussion

To evaluate the patient’s comfort after the surgery of the impacted 3 M, degree of pain, swelling, and trismus with the presence of complications, such as bleeding, wound healing abnormalities, alveolitis, and infections are usually questioned. In previous studies, the effectiveness of any additional or different procedure on postoperative morbidity has been measured by using these parameters. The present study aimed to reveal the effects of primary and secondary wound healing and primary healing using collagen membranes in terms of these variables.

There was no significant difference between the groups regarding degree of pain in this study, except for the lower pain degree reported in the SC group compared to the MBPC group on postoperative day 2. This finding is similar to the findings of various studies comparing wound closures with or without drain and partial and total closures [2, 14–17]. However, some studies reported less pain in partial closure and wound closure with drain [1, 18–21]. Pain increases with increasing difficulty of surgery, notably in operations requiring the raising of a mucoperiosteal flap [13]. In a study on the partially impacted mandibular 3 M, in appropriate cases, Kim et al. [22] compared buccal flap and flapless procedures and reported extremely low degrees of pain in the flapless procedure. In the present study, there was generally no significant difference in the degree of pain between the groups, which may be due to the fact that the flap type is standardized, and there was no difference in the degree of surgical difficulty between the groups. At this point, it can be considered that the flap raising or tissue damage is a more effective factor in the formation and severity of pain, rather than wound closure type. Our results indicate that pain evaluated using the VAS was most severe on the day of surgery in all groups, as expected, and this was in line with previous studies. Moreover, pain subsequently declined steadily until postoperative day 7.

In this study, mouth opening inability and swelling were found to be less severe in the SC group, particularly in the postoperative immediate period. In accordance with our findings, Rakprasitkul and Pairuchvej [23], investigated the effect of the use of a small surgical tube drain together with primary wound closure, as compared to primary wound closure alone, after removal of impacted mandibular 3Ms. They reported significantly wider mouth opening in the immediate postoperative period and significantly less facial swelling in the drain group, and there was no significant difference in the severity of pain between the two groups. When the findings of other studies evaluating types of wound closure were viewed, some studies [16, 17] have indicated that there is no difference between the two groups in terms of these parameters, and in some studies [1, 18, 19, 24], less pain and swelling rates were reported in the secondary healing group. Further, in terms of swelling alone, some studies [2, 14, 15] have reported lower rates in the secondary group. In a study comparing the effects of total and partial wound closure techniques on immediate postoperative tissue reactions and complications after mandibular 3 M surgery, Bello et al. [2] reported that a statistically significant reduction in facial swelling was found in partial closure, but no difference was recorded for pain and trismus.

Extent of swelling may affect the patient’s social and working life, and it is one of the indicators of comfort during the postoperative period [1, 21]. Both objective and subjective methods were used to evaluate swelling in the present study. Both methods have also been used in previous investigations to determine the degree of swelling [1, 2, 23, 24]. Objective measurements provide clear information to clinicians in determining the efficacy of any treatment, but patient-centered thinking and consideration of patients’ perceptions may become even more important in procedures where patient comfort is assessed. Hence, both methods are included in the current study. In the present study, there was no significant difference between the PC and MBPC groups in the objective measurement method, whereas in the subjective method, lower swelling was found in the PC group than the MBPC group. Swelling could be due to accumulation of inflammatory exudate within facial tissues [19], hematoma collection [25], or both [2]. In the present study, the fact that the secondary closure was significantly superior to the primary closure in terms of swelling could be explained with the well-accepted view in the literature that partial wound closure supplies drainage and minimizes immediate postoperative edema [2, 16, 18] minimizes immediate postoperative edema [2, 16, 18].

In this study, there were no significant differences on the 7^th^ postoperative day in the amount of mouth opening restriction, which is a trismus indicator, while significant differences were observed on the 2^nd^ postoperative day. Consistent with this finding, in their study comparing primary and secondary closure, in terms of trismus, Singh et al. [26] found the secondary closure group to be significantly less restricted on the 3^rd^ postoperative day, and no difference was found between the two groups on the 7^th^ postoperative day. However, Bello et al. [2] reported no differences on either the 2^nd^ or 7^th^ postoperative day between the two groups. Trismus after mandibular 3 M surgery is usually caused by inflammation of the masticatory muscles, leading to spasm secondary to the raising of a mucoperiosteal flap [4, 27]. In this regard, the SC group may have been less restricted in this study due to the effect of drainage. The restriction was also found to be greatest in the MBPC group, which may have been triggered due to the increased tension of the flap via the membrane in addition to flap sliding.

In the present study, the postoperative bleeding rate was 0%, as no bleeding was found in any of the groups. Consistent with this finding, Pasqualini et al. [1] reported an incidence of 0%. In a study aiming to identify the types, frequency, and risk factors for complications after 3 M extractions, Bui-Chi et al. [28] reported a fairly low postoperative bleeding rate of 1.2%, which they defined as any bleeding that might require extra treatment. On the other hand, Bello et al. [2] reported a rate as high as 24.4%.

Wound dehiscence was found in 20% of the PC group and 6.7% in the MBPC group, and there was no significant difference between the two groups. In the present study, the relatively high dehiscence ratio in the primary healing group may be due to the increased tension of the sliding flap. Sandhu et al. [29] and Jakse et al. [7], who applied vestibular triangular flap types, reported relatively low rates of 5% and 10%, respectively. However, in a study applying the same flap type, Pasqualini et al. [1] reported a rate of dehiscence as high as 33% distal to the 2 M. In the studies mentioned above, it has been noticed that the teeth included these studies were completely covered with mucosa. In such cases are superior in terms of primary healing because of there is no tissue loss when the flap is closed to its original position after surgery [3, 7]. The relatively high dehiscence ratio (33%) reported by Pasqualini et al. [1] may be due to study variables differences between this study and our study. On the other hand, relatively minimal wound dehiscence in the membrane-based primary closure group suggests that resorbable collagene membrane usage may support primary healing.

In the present study, there was no statistically significant difference in the incidence of dry socket between the groups. This finding is consistent with the findings of other studies [1, 2, 18]. Similarly, Danda et al. [24] compared the influence of primary and secondary closure of the surgical wound on alveolitis after removal of impacted mandibular 3Ms and recorded no difference between these 2 groups. It has been claimed that the primary wound closure may be susceptible to dry socket because it cannot completely clean itself like a normal alveolar socket [18]. However, no clear information could be obtained that revealed the weakness of the primary closure in this respect. Further, there was no alveolitis in the MBPC group. This may be due to the membrane’s contribution to the clot stabilization and the isolation.

In the present study, socket infection was not encountered in any of the cases during the 4-week follow-up period. There were also no patients who subsequently presented with signs of infection, despite the warning in this regard. In a study investigating the incidence of socket infection in the primary and secondary closure, the 2 cases of socket infection Bello et al. [2] recorded in their study were associated with third molars in the primary wound closure group, despite the statistically insignificant difference between the 2 groups. Similar to this, Pasqualini et al. [1] reported that primary closure was complicated by suppurative alveolitis that originated from the periodontal pocket distal to the 2 M 3 or 4 weeks after surgery in 2% of cases in this series. Previous investigations have reported a low (1.5–1.6%) incidence of postoperative late period infection [5, 30]. In a study in which collagen and biodegradable membrane types were compared in the extraction of bilateral fully impacted molars in the same procedure with the split mouth method in terms of bone regeneration in 15 patients, Zwahlen et al. [31] reported complications such as wound infection and hematoma with pus formation and late swelling 10 days after surgical removal molar occurred in 3 patients.

## Conclusion

In conclusion, according to the results of the present study, the secondary closure provides a marked advantage over the primary closure in terms of swelling and mouth opening. Primary closure and primary closure using the collagen membrane are relatively similar in terms of immediate postoperative discomfort. As the primary outcome of this study, resorbable collagene membrane usage showed clinically satisfactory results. Additionally, the absence of alveolitis in the primary closure using the collagen membrane and minimal wound dehiscence, suggests that membrane usage may support primary healing in terms of wound healing.

## Abbreviations

2 M: second molar tooth
3 M: Third molar tooth
MBPC: Membrane based primary closure
OPG: Orthopantomography
PC: Primary closure
SC: Secondary closure
VAS: Visual analog scale
